# Effect of Synthesis Factors on Microstructure and Thermoelectric Properties of FeTe_2_ Prepared by Solid-State Reaction

**DOI:** 10.3390/ma16227170

**Published:** 2023-11-15

**Authors:** Lang Zhang, Bingke Qin, Cheng Sun, Yonghua Ji, Dan Zhao

**Affiliations:** 1 College of Physical Science and Technology, Dalian University, Dalian 116622, China; 17718171252@163.com; 2 School of Chemistry and Materials Engineering, Liupanshui Normal University, Liupanshui 553004, China; haizhigong@126.com (Y.J.); zhaodan0910@126.com (D.Z.)

**Keywords:** thermoelectric properties, FeTe_2_, microstructure, phase composition

## Abstract

The alloying compound FeTe_2_ is a semi-metallic material with low thermal conductivity and has the potential to become a thermoelectric material. Single-phase FeTe_2_ compounds are synthesized using a two-step sintering method, and the effects of the optimal sintering temperature, holding temperature, and holding time on the thermoelectric properties of the alloy compound FeTe_2_ are investigated. The phase composition, microstructure, and electrical transport properties of the FeTe_2_ compound are systematically analyzed. The results show that single-phase FeTe_2_ compounds can be synthesized within the range of a sintering temperature of 823 K and holding time of 10~60 min, and the thermoelectric properties gradually deteriorate with the prolongation of the holding time. Microstructural analysis reveals that the sample of the alloy compound FeTe_2_ exhibits a three-dimensional network structure with numerous fine pores, which can impede thermal conduction and thus reduce the overall thermal conductivity of the material. When the sintering temperature is 823 K and the holding time is 30 min, the sample achieves the minimum electrical resistivity of 6.9 mΩ·cm. The maximum Seebeck coefficient of 65.48 μV/K is obtained when the sample is held at 823 K for 10 min; and under this condition, the maximum power factor of 59.54 μW/(m·K^2^) is achieved. In the whole test temperature range of 323~573 K, when the test temperature of the sample is 375 K, the minimum thermal conductivity is 1.46 W/(m·K), and the maximum *ZT* is 1.57 × 10^−2^.

## 1. Introduction

Recently, the environmental pollution caused by the excessive consumption of non-renewable energy has become increasingly serious, which has attracted widespread attention worldwide [1]. To alleviate the potential energy crisis and environmental pollution in the future, it is crucial to explore eco-friendly and sustainable new energy sources and technologies [2,3]. Thermoelectric materials are energy-harnessing solid-state semiconductors that transform thermal energy into electricity or create a temperature difference from an applied voltage [4]. The thermoelectric conversion effect can be used to make a thermoelectric generator that converts heat energy into electrical energy, and can also be used to make a refrigerator that uses electrical energy directly for cooling [5,6,7]. A device made of thermoelectric materials consists of n-type and p-type thermoelectric materials connected to electrodes to form a p-n junction in series or parallel. These devices offer several advantages including no moving parts, light weight, small size, no need for refrigerants, no pollution, and long service life [1,2,8]. Therefore, in today’s world, where environmental pollution and energy crises are constantly threatening the survival of mankind, the research on thermoelectric materials holds significant practical significance.

The performance of TE materials is measured by the dimensionless figure of merit, *ZT*, given by *ZT* = *S*^2^*σT*/*κ*, where *σ*, *S*, *T*, and *κ* represent the electrical conductivity, Seebeck coefficient, operating temperature, and thermal conductivity, respectively [9,10,11,12,13,14,15,16,17,18,19,20]. Both the cooling and power generation efficiency of thermoelectric materials are functions of the *ZT* value, with higher *ZT* values indicating a higher efficiency. From the formula, it can be seen that there are two ways to improve the thermoelectric performance: increasing the power factor (*σS*^2^) or reducing the thermal conductivity. The total thermal conductivity (*κ_tot_*) is composed of the lattice thermal conductivity (*κ_lat_*) and electronic thermal conductivity (*κ_ele_*), *κ_tot_* = *κ_lat_* + *κ_ele_* [21,22,23]. *κ_ele_* is the electronic thermal conductivity calculated using the Wiedemann–Franz law *κ_ele_* = *LσT*, where *L* is the Lorenz constant, *T* is the absolute temperature, and *σ* is proportional to the electrical conductivity. *κ_lat_* is the lattice thermal conductivity caused by the vibrational motion of phonons. For most materials, the three parameters, Seebeck coefficient, electrical resistivity, and thermal conductivity, which affect the thermoelectric properties of the material are not independent of each other. They all depend on the electronic structure of the material as well as carrier transport and scattering. *S* decreases with the increase in the carrier concentration *n*, while *σ* is positively correlated with *n*. If these two parameters are properly balanced, the power factor can be optimized. In addition, *κ* can be reduced by forming point defects or adjusting the structural properties, such as atomic defect, dispersed particle, grain boundary, and nanostructuring [24,25]. Therefore, scientists tend to search for materials with high Seebeck coefficients, expecting to reduce their thermal conductivity while improving their electrical conductivity to obtain higher *ZT* values.

Transition metal chalcogenides have received increasing attention as a potential electronic material due to their tunable electrical transport properties. Transition metal chalcogenides have a high density of states owing to the d and f orbital electrons of transition metals, which can induce a high power factor [16]. Kim et al. [26] doped 2% Br in SnSe_2_ and achieved a maximum *ZT* 0.62 at 750 K, which is 50% higher than the undoped SnSe_2_. Pang et al. [27] optimized their thermoelectric properties by Pb and I co-doped SnTe, reaching a *ZT* of 0.8 at 573 K. Recently, Fe-based chalcogenides FeTe_2_, which have an orthorhombic structure, have been suggested as good candidates for thermoelectric applications [28]. The current research is mainly used for solar cell materials and lithium battery electrode materials, but there are few reports that it has been studied as a thermoelectric material. Through experimental exploration, it has been found that FeTe_2_ has a larger Seebeck coefficient and appropriate resistivity compared with NiTe_2_ and CoTe_2_, which has certain research value. The thermoelectric performance of the sample can be optimized by doping or as a second phase for composites with other thermoelectric materials. Yang et al. [9] found that the introduction of 1% FeTe_2_ into Ge_0_._9_Sb_0_._1_Te can obtain a maximum *ZT* value of 2.1 at 723 K, which greatly improves the thermoelectric performance.

**Figure 1:**
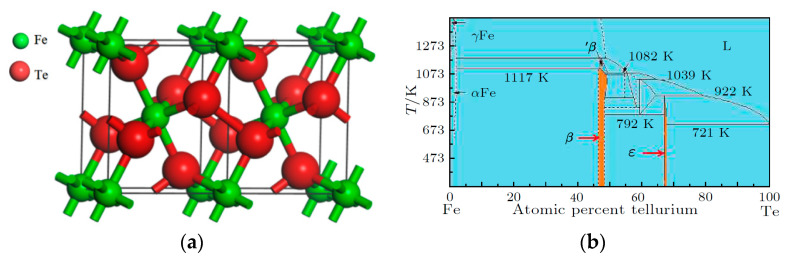
(**a**) The lattice structure of FeTe_2_; (**b**) the binary phase diagram of Fe-Te [39].

The marcasite-type compound FeTe_2_ belongs to the orthorhombic crystal system with the space group *Pnnm* and lattice constants *a* = 0.526 nm, *b* = 0.626 nm, and *c* = 0.386 nm [10,29]. Its crystal lattice structure is shown in Figure 1a; each iron atom is coordinated by six tellurium atoms, and each tellurium atom is also coordinated by three iron atoms. Depending on the synthesis conditions, FeTe_2_ can form either a marcasite or pyrite crystal structure. The crystal phases formed at room temperature are mostly orthorhombic martensitic structures, while those formed at high temperatures and high pressures are cubic pyrite structures [30]. The crystal structure of FeTe_2_ exhibits a high symmetry, narrow band gap, three-dimensional magnetic order, and semiconductor properties [31,32], making it a potential semiconductor thermoelectric material. Recently, FeTe_2_ has shown special properties in areas such as magnetism [33,34], optics [35], and catalysis [36,37]. Traditional methods for synthesizing FeTe_2_ include chemical vapor transport [38], hydrothermal methods [32], and a high-pressure preparation method [29]. Other preparation methods require relatively long synthesis times, except for high pressure, which requires a short synthesis time but expensive equipment. High-temperature solid-state reaction sintering can not only synthesize samples within 3~6 h, but can also obtain 6~10 samples at a time by “one pot multi-burning”, which greatly reduces the preparation cost and facilitates parallel repeated experiments. Compared with the traditional preparation method, it has great advantages. In this experiment, FeTe_2_ is prepared by the two-step sintering method, and its phase structure and electrical properties are analyzed.

## 2. Experiment

### 2.1. Sample Preparation

The starting materials used in the experiment were Fe powder and Te powder with a purity of 99.99% and a particle size of approximately 300 mesh. The starting materials were accurately weighed according to the stoichiometric ratio of the chemical formula FeTe_2_. They were then thoroughly ball-milled under an inert atmosphere for 2 h, with a ball-to-powder ratio of 30:1 and a rotation speed of approximately 350 r/min using a planetary ball mill. The milled powders were cold-pressed into cylindrical pellets with a diameter of 12 mm and a height of 3.5 mm under a pressure of 3~5 MPa. Subsequently, the pellets were sealed in the sintering mold and placed in a vacuum furnace synthesis. The temperature was 723~923 K, the intermediate holding temperature was 673~763 K, the first holding time was 30~180 min, and the second holding time was 10~60 min. After cooling to near room temperature, the samples were removed from the mold and polished with sandpaper to remove surface oxidation or uneven parts. The samples were then subjected to ultrasonic cleaning, dried, and tested for XRD, SEM, Seebeck coefficient, thermal conductivity, and electrical resistivity.

### 2.2. Sample Testing and Characterization

The vacuum furnace model used for solid-state reaction sintering was HMZ-1700-20 (Shanghai Haoyue high-temperature equipment Co., Ltd., Shanghai, China), and the phase composition analysis of the sample was performed using a TD-2500 X-ray diffractometer (Dandong Tongda Technology Co., Ltd., Dandong, China) with a scanning step size of 0.02° and a diffraction angle range of 20~80°. The microstructure of the sample was tested using an FEI Nova Nano SEM 450 ultra-high-resolution scanning electron microscope (FEI, Hillsboro, OR, USA). The electrical resistivity of the sample at room temperature was measured using an RTS-9 four-probe tester (Guangzhou four probe technology Co., Ltd., Guangzhou, China), and the Seebeck coefficient was tested using a self-made calibrated Seebeck tester [40]. The *κ* was calculated using *κ* = *D*·*C_P_*·*ρ*, where *D* was the thermal diffusivity measured on an LFA457 laser flash unit, *C_P_* estimated from the Dulong–Petit law was the heat capacity, and *ρ* measured by the Archimedes’ principle was the density.

## 3. Results and Discussion

### 3.1. Phase Composition and Micromorphology Analysis

**Figure 2:**
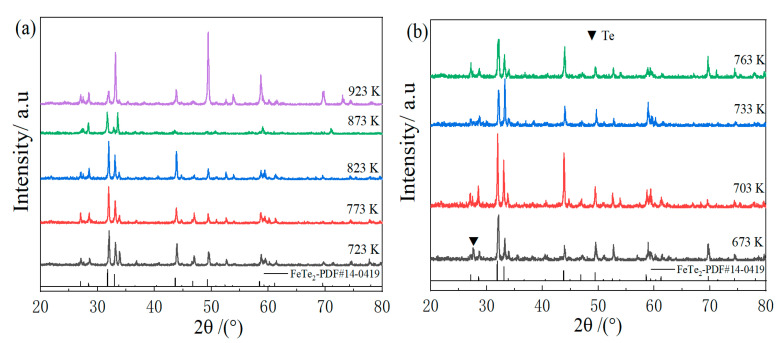

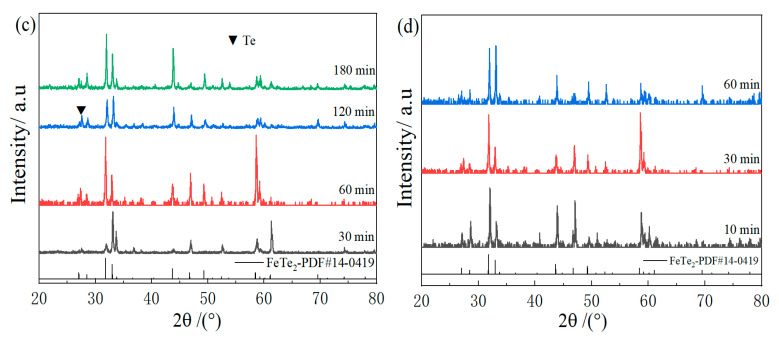
XRD patterns of samples under different preparation conditions: (**a**) XRD patterns at different synthesis temperatures; (**b**) XRD patterns at different intermediate holding temperatures; (**c**) XRD pattern under different holding times in the previous period; (**d**) XRD pattern under different holding times in the latter period.

The XRD pattern of the FeTe_2_ sample prepared by solid-state reaction is shown in Figure 2a. From the figure, it can be observed that the peak positions and relative peak intensities of the samples in the range of 723~873 K are consistent with the standard diffraction card PDF#14-0419 data for FeTe_2_. At 923 K, the crystallinity of the sample significantly improved, but there are numerous pores in the sample, which may be due to the high sintering temperature causing volatilization of the Te element. Additionally, the growth rate of certain crystal planes increased significantly, indicating the possibility of other phases with similar nominal compositions, but the appearance of “solid solution”-like phases might also have appeared. Figure 2b shows the exploration of the intermediate holding temperatures, and it can be seen that single-phase FeTe_2_ compounds are synthesized at temperatures of 703~763 K. When the temperature is 673 K, there is noticeable precipitation of elemental Te, possibly due to insufficient initial kinetic energy resulting in incomplete reaction. Figure 2c displays the exploration of the first holding time. Single-phase FeTe_2_ compounds are synthesized when the time is 30~60 min and 180 min. At 120 min, precipitation of elemental Te occurred, possibly due to the decomposition of FeTe_2_ into elemental Te with the prolonged holding time. With a further extension of the holding time, the volatile elemental Te disappeared, and the single-phase FeTe_2_ compound is still obtained with a holding time of 180 min. However, if a material can be synthesized at a given temperature, which needs to react the components with each other, the increase in the reaction time should not decrease the yield. Therefore, the increase in the content of elemental tellurium at this time is unexpected. From the phase diagram of the Fe-Te system, as shown in Figure 1b, it can be seen that there are two main phases between Fe and Te: β(FeTe), ε(FeTe_2_), and the product is the ε-phase of FeTe_2_ when Fe:Te = 1.9~2.1 [41,42]. Combined with Figure 2c, it can be seen that the peak intensities at specific ratios of Fe and Te vary with certain parameters (temperature/time), which indicates the formation of more than one phase with similar crystallographic parameters. Therefore, the formation of a Te-rich phase at a given into a Te-poorer phase and elemental Te on keeping the sample at a given temperature. Figure 2d represents the exploration of the second holding time. It can be observed that single-phase FeTe_2_ compounds are synthesized at 10~60 min. However, as the holding time increased, more pores appeared in the sample, leading to a decrease in the thermoelectric properties with time.

**Figure 3:**
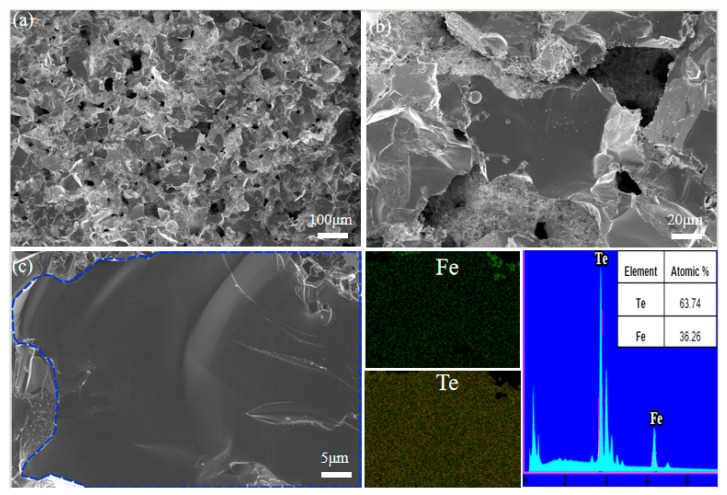
SEM and EDS image of section plane inner of FeTe_2_: (**a**) lower magnification; (**b**) higher magnification; (**c**) EDS mapping analysis.

Figure 3 shows the scanning electron microscope (SEM) micrograph and energy-dispersive X-ray spectroscopy (EDS) spectrum of the cross-section of the FeTe_2_ sample. Figure 3a has a lower magnification, while Figure 3b has a higher magnification. From the figure, it can be observed that the sample has a three-dimensional network structure with unevenly distributed pores. These pores are likely formed during the solid-phase reaction synthesis process when different components of the raw materials undergo interface reactions and fuse into a new phase. This complex micro-porous structure can obstruct the heat transfer effect, thereby reducing the overall thermal conductivity of the material. Figure 3c shows the EDS mapping analysis results. Due to factors such as sample pores and uneven cross-sections, the mapping distribution in the spectrum is not uniform. However, on the same relatively smooth grain surface, the distribution of the elements Fe and Te is uniform. In the elemental spectrum analysis, no other impurity elements are found besides Fe and Te, indicating that the sample prepared by the solid-phase reaction method does not contain significant impurities.

### 3.2. Analysis of Thermoelectric Properties

**Figure 4:**
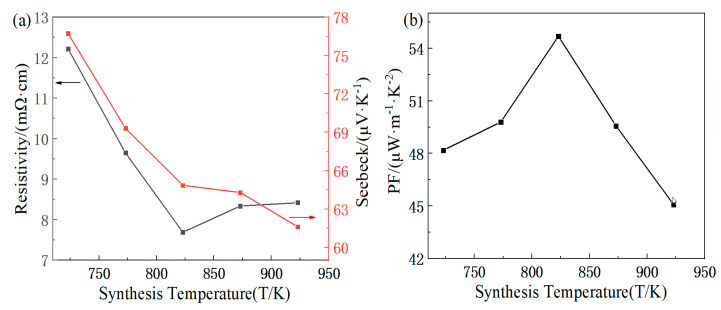
Synthesis temperature dependence of electricity properties for FeTe_2_: (**a**) electrical resistivity, Seebeck coefficient; (**b**) power factor.

The variation in the electrical resistivity and Seebeck coefficient with the synthesis temperature for the FeTe_2_ sample at room temperature is shown in Figure 4a. The Seebeck coefficient of the sample is positive, which is a p-type semiconductor. For metals or degenerate semiconductors, assuming that carrier scattering is independent of the temperature, the Seebeck coefficient can be expressed as:

$$S=\frac{8\pi^{2}{K_{B}}^{2}}{3eh^{2}}m^{*}T{\left(\frac{\pi }{3n}\right)}^{\frac{2}{3}}$$

where *K_B_* is the Boltzmann constant, *e* is the elementary charge, *h* is the Planck constant, *T* is the absolute temperature, *n* is the carrier concentration, and m* is the effective mass of the carriers. The Seebeck coefficient is directly proportional to the effective mass of the carriers and inversely proportional to the 2/3 power of the carrier concentration. In general, for metallic thermoelectric materials, the higher the carrier concentration and carrier mobility, the smaller the Seebeck coefficient. With the increasing sintering temperature, the electrical resistivity initially decreases and then increases. During the synthesis process of FeTe_2_, the element Te is prone to volatilization, leading to an excess of Fe entering the interstitial sites in the crystal lattice and providing additional electrons as carriers. Therefore, the Seebeck coefficient decreases with the increase in the carrier concentration. The Seebeck coefficient of 76.74 μV/K is achieved at around 723 K, and the electrical resistivity reaches a value of 7.69 mΩ·cm at 823 K. Figure 4b shows the variation trend of the Seebeck coefficient of the sample with the preparation temperature. It can be seen from the figure that the power factor presents a law of first increasing and then decreasing, showing a parabolic variation trend, and the maximum value can be obtained. At 823 K, the maximum power factor is 54.70 μW/(m·K^2^).

**Figure 5:**
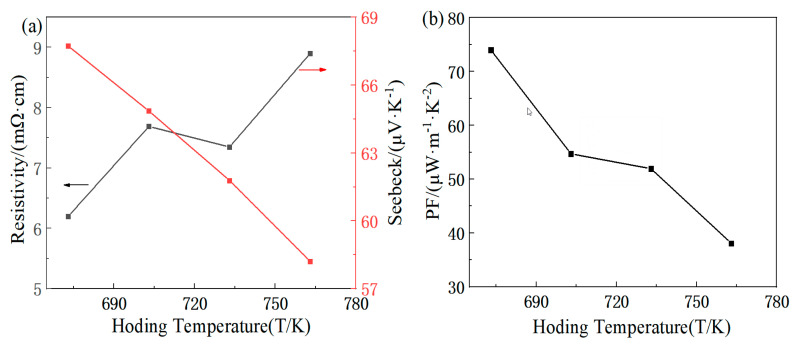
Holding temperature dependence of electricity properties for FeTe_2_: (**a**) electrical resistivity, Seebeck coefficient; (**b**) power factor.

Figure 5a shows the variation in the electrical resistivity and Seebeck coefficient with the intermediate holding temperature. Since the melting point of Te is around 723 K, four points within the range of 673~763 K are selected to explore the intermediate temperature. With the increase in the temperature, the electrical resistivity increases while the Seebeck coefficient decreases. This may be due to the volatilization of the Te element caused by the temperature rise, the internal pores increase the resistivity, and the thermal excitation increases the carrier concentration of the sample, resulting in the decrease in the Seebeck coefficient. Under the condition of synthesizing single-phase FeTe_2_, the electrical resistivity of 7.35 mΩ·cm is achieved at around 733 K, and the Seebeck coefficient reaches a value of 64.89 μV/K at 703 K. Figure 5b illustrates the variation in the power factor with the intermediate holding temperature. As the temperature increases, the power factor tends to decrease continuously. Under the condition of synthesizing the FeTe_2_ compound, the maximum power factor of 54.70 μW/(m·K^2^) is achieved at 703 K.

**Figure 6:**
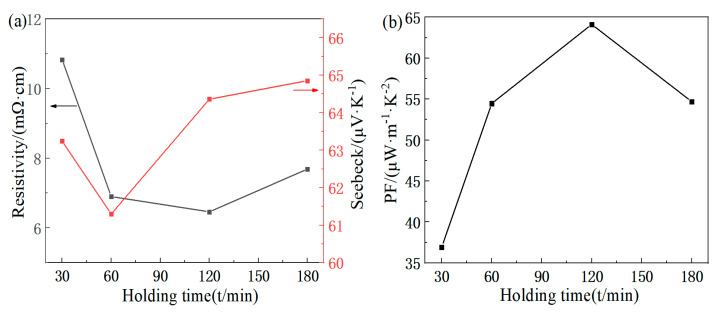
First holding time dependence of electricity properties for FeTe_2_: (**a**) electrical resistivity, Seebeck coefficient; (**b**) power factor.

The variation in the electrical resistivity and Seebeck coefficient with the first holding time is shown in Figure 6a. As the time increases, both the electrical resistivity and Seebeck coefficient initially decrease and then increase. At 120 min, there is noticeable precipitation of elemental Te, and it is found that trace amounts of elemental Te precipitation actually benefit the thermoelectric properties of FeTe_2_. Under the condition of synthesizing the single-phase FeTe_2_ compound, the electrical resistivity of 6.9 mΩ·cm is achieved at 60 min, and the Seebeck coefficient of 64.86 μV/K is achieved at 180 min. Figure 6b illustrates the variation in the power factor with the preceding holding time. From the figure, it can be observed that the power factor initially increases and then decreases as the time is extended. Considering the energy-saving factors, there is not much difference in the power factor between 60 min and 180 min. Therefore, the optimal condition for maximizing the power factor is determined to be a holding time of 60 min, achieving a power factor of approximately 54.46 μW/(m·K^2^).

**Figure 7:**
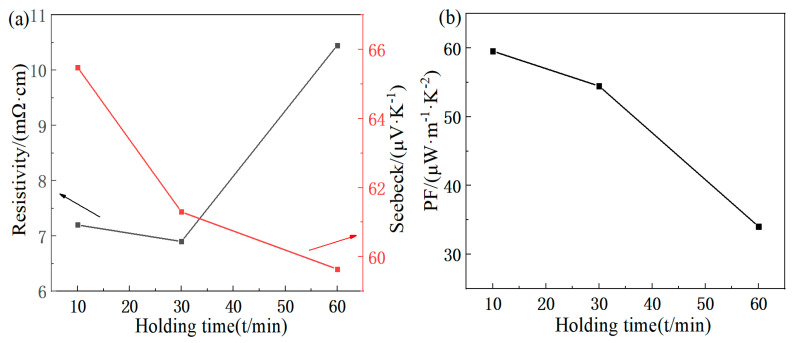
Second holding time dependence of electricity properties for FeTe_2_: (**a**) electrical resistivity, Seebeck coefficient; (**b**) power factor.

The variation in the electrical resistivity and Seebeck coefficient with the second holding time is shown in Figure 7a. As the time increases, the electrical resistivity decreases while the Seebeck coefficient increases. The electrical resistivity of 6.9 mΩ·cm is achieved at 30 min. At 10 min, the Seebeck coefficient of 65.48 μV/K is obtained. Figure 7b shows that the power factor decreases with time, and the holding time of 10 min is selected, resulting in a maximum power factor of 59.54 μW/(m·K^2^).

In the following, the variation rule of the alloy compound FeTe_2_ is examined to test the thermoelectric properties at different temperatures, and based on the results of the optimal thermoelectric properties at room temperature, the samples with the preparation temperature of 823 K are selected for the variable temperature test. The first temperature point of the temperature-dependent test is around 323 K. At this temperature, the obtained electrical resistivity for the FeTe_2_ sample is 6.42 mΩ·cm, and the Seebeck coefficient is 66.79 μV/K. Compared to room temperature, the electrical resistivity decreased by approximately 10%, while the Seebeck coefficient increased by about 2%. This indicates that the electrical property results obtained by our research group’s equipment are relatively accurate, although generally slightly lower than commercially available values.

**Figure 8:**
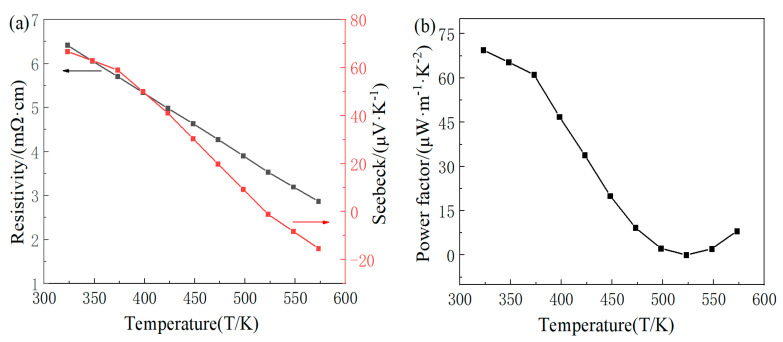
Relation of electrical properties of FeTe_2_ block to test temperature: (**a**) electrical resistivity, Seebeck coefficient; (**b**) power factor.

Figure 8a displays the variation in the electrical resistivity and Seebeck coefficient of the FeTe_2_ sample with respect to the testing temperature. From the figure, it can be observed that within the temperature range of the test, the Seebeck coefficient changes from positive to negative, indicating a transition from p-type to n-type semiconductor behavior, and the carrier changes from hole to electron. Moreover, as the temperature increases, the electrical resistivity of the sample continues to decrease, while the absolute value of the Seebeck coefficient exhibits a trend of initially decreasing and then increasing. The minimum electrical resistivity of 2.87 mΩ·cm is achieved at 573 K, and the Seebeck coefficient reaches a maximum value of 66.79 μV/K at around 323 K.

The power factor of the sample is shown in Figure 8b. The power factor is calculated by the Seebeck coefficient and electrical resistivity. Due to its bipolar nature, FeTe_2_ exhibits the ability to have both p-type and n-type semiconductor characteristics. This means that under different conditions, FeTe_2_ can demonstrate conduction behavior with positive charge carriers (holes) and negative charge carriers (electrons). As can be seen from Figure 8, the Seebeck coefficient changes from positive to negative over the entire test temperature range, although the electrical resistivity continues to decrease. This change causes the Seebeck coefficient to decrease in the positive direction and then increase in the reverse direction, which leads to the decrease in the power factor with the increase in the temperature and then increase. The maximum power factor obtained at 323 K is 69.46 μW/(m·K^2^).

**Figure 9:**
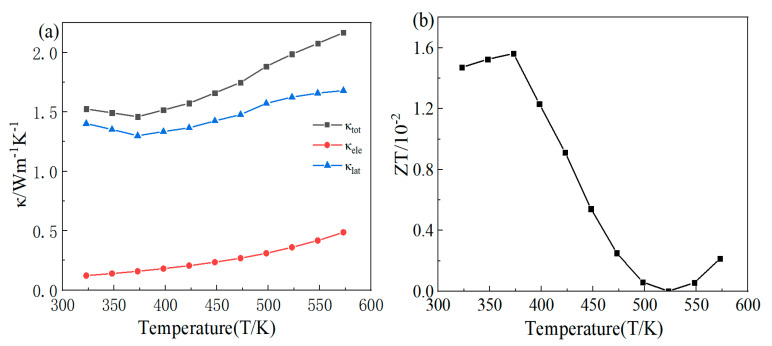
Relation of thermal properties, *ZT* of FeTe_2_ block to test temperature: (**a**) total thermal conductivity, lattice thermal conductivity, thermal conductivity of electrons; (**b**) thermoelectric value *ZT*.

Figure 9a shows the variation in the total thermal conductivity, lattice thermal conductivity, and electronic thermal conductivity of the FeTe_2_ sample prepared by the solid-state reaction method with respect to the test temperature. From the figure, it can be observed that at room temperature, the total thermal conductivity and lattice thermal conductivity of the FeTe_2_ sample are relatively close. This is due to the low carrier concentration in the sample at room temperature, resulting in a smaller electronic thermal conductivity. Subsequently, as the temperature continues to increase, both the total thermal conductivity and lattice thermal conductivity exhibit a similar trend of initially decreasing and then increasing. The electronic thermal conductivity arises from the heat conduction effect caused by the motion of electrons and holes in the sample. Therefore, as the temperature increases, thermal excitation leads to an increase in the carrier concentration, resulting in a continuous increase in the electronic thermal conductivity. Due to the existence of many micro-pores, the overall thermal conductivity of the FeTe_2_ samples is relatively small. At 373 K, the FeTe_2_ sample obtains the minimum total thermal conductivity of 1.46 W/(m·K).

The relationship between the *ZT* value of the FeTe_2_ sample and the test temperature is shown in Figure 9b, and its *ZT* value is calculated based on the test results of the electrical and thermal properties. When the temperature is less than 373 K, the *ZT* value increases continuously, and reaches a maximum value of 1.57 × 10^−2^ at 373 K. At 373~523 K, the Seebeck coefficient transitions from p-type to n-type semiconductors, resulting in a gradual decrease in the *ZT* values towards zero. At 523 K, the Seebeck increases reversely, resulting in a slight increase in the *ZT*. In general, it can be seen that devices composed of FeTe_2_ are more suitable for use at room temperature.

## 4. Conclusions

Overall, the optimal conditions for maximizing the power factor are determined as follows: synthesis temperature of 823 K, intermediate holding temperature of 703 K, first holding time of 60 min, and second holding time of 10 min. The power factor reaches a maximum value of 59.54 μW/(m·K^2^).

Throughout the entire test temperature range, the minimum electrical resistivity obtained is 2.87 mΩ·cm at 573 K. The maximum Seebeck coefficient of 66.79 μV/K is achieved at 323 K, along with the highest power factor of 69.46 μW/(m·K^2^). The minimum thermal conductivity is achieved at 373 K, measuring only 1.46 W/(m·K), and a maximum figure of merit value of 1.57 × 10^−2^.

## Data Availability

The data presented in this study are available on request from the corresponding author. The data are not publicly available due to technical or time limitations.
